# Experience of After-Effect of Memory Update Reduces Sensitivity to Errors During Sensory-Motor Adaptation Task

**DOI:** 10.3389/fnhum.2021.602405

**Published:** 2021-03-15

**Authors:** Kenya Tanamachi, Jun Izawa, Satoshi Yamamoto, Daisuke Ishii, Arito Yozu, Yutaka Kohno

**Affiliations:** ^1^Graduate School of Health Sciences, Ibaraki Prefectural University of Health Sciences, Ibaraki, Japan; ^2^Faculty of Engineering, Information and Systems, University of Tsukuba, Ibaraki, Japan; ^3^Department of Physical Therapy, Ibaraki Prefectural University of Health Sciences, Ibaraki, Japan; ^4^Center of Medical Science, Ibaraki Prefectural University of Health Sciences, Ibaraki, Japan; ^5^Department of Cognitive Behavioral Physiology, Chiba University Graduate School of Medicine, Chiba, Japan; ^6^Department of Precision Engineering, The University of Tokyo, Tokyo, Japan

**Keywords:** error-sensitivity, sensory-motor adaptation, motor learning, after-effect, error related negativity

## Abstract

Motor learning is the process of updating motor commands in response to a trajectory error induced by a perturbation to the body or vision. The brain has a great capability to accelerate learning by increasing the sensitivity of the memory update to the perceived trajectory errors. Conventional theory suggests that the statistics of perturbations or the statistics of the experienced errors induced by the external perturbations determine the learning speeds. However, the potential effect of another type of error perception, a self-generated error as a result of motor command updates (i.e., an aftereffect), on the learning speeds has not been examined yet. In this study, we dissociated the two kinds of errors by controlling the perception of the aftereffect using a channel-force environment. One group experienced errors due to the aftereffect of the learning process, while the other did not. We found that the participants who perceived the aftereffect of the memory updates exhibited a significant decrease in error-sensitivity, whereas the participants who did not perceive the aftereffect did not show an increase or decrease in error-sensitivity. This suggests that the perception of the aftereffect of learning attenuated updating the motor commands from the perceived errors. Thus, both self-generated and externally induced errors may modulate learning speeds.

## Introduction

Sensorimotor adaptation is a process through which the brain updates motor commands after perceiving a trajectory error generated by a perturbation. Interestingly, the brain has a great capability to accelerate learning over several training sessions. For instance, the speed of learning during the 2nd training session is often faster than that during the 1st session, a phenomenon that is called “savings” (Krakauer et al., 1999; Kojima et al., 2004). Representative computational theory of motor adaptation suggests that this acceleration is influenced by the statistics of perturbations: when the direction of the perturbation is consistent, the speed of learning increases over sessions, whereas when the direction of the perturbation changes often, the speed decreases (Gonzalez et al., 2014). This is in line with the Kalman filtering theory of motor adaptation, where learning speed is characterized by the noise in the environment (Burge et al., 2008). These computations might be achieved by the cortical representation of sensory prediction errors (Herzfeld et al., 2014); when an experienced error is relatively consistent, the speed of learning should increase over training sessions. One limitation of these models is that they focus only on errors induced by external perturbations such as force fields or visual rotations. Since trajectory errors are also generated by variabilities in representations of motor commands (Van Beers et al., 2004), such a mechanism of learning acceleration can also be influenced by self-generated errors. However, whether self-generated errors regulate learning speed in the same way as externally generated errors has not been examined yet.

Here, we focused on the self-generated errors induced by aftereffects during memory updates. For example, when a participant updates their motor memory in response to a force perturbation and then subsequently experiences a zero-force (Null) environment, they should perceive trajectory errors in the Null environment, which are generated as a result of the aftereffect of the motor command update. If the external perturbation statistics are the only cause of the modulation of the learning speed, i.e., sensitivity to the error in the memory update, the experience of the self-generated errors should not alter the participant’s error-sensitivity. Alternatively, if the self-generated errors induced by the aftereffects provide essential information for the error-sensitivity adjustments, the exposure to the self-generated errors may alter the participant’s learning speeds.

To test this prediction, we had all participants experience the force field perturbations, with one group perceiving aftereffects of memory updates as trajectory errors in a Null environment and the other group not perceiving the aftereffects as the trajectory error that was clamped in a channel-force (Channel) environment. Here, we found the attenuated error-sensitivity only in the group who perceived the aftereffect. We concluded that the self-generated errors as a result of memory updates modulated learning speed.

## Materials and Methods

### Participants

A total of 20 healthy right-handed volunteers (11 males and nine females; 20–35 years old) participated in our experiments after providing signed informed consent. The participants had no known neurological and skeletal problems, were naïve to the purpose of the experiments, and were randomly divided into two groups: the FF-Null group (*n* = 10) and the FF-Channel group (*n* = 10). The protocol of the experiment was approved by the Ibaraki Prefectural University of Health Sciences Review Board and the University of Tsukuba Ethics Committee, which was conducted in conformity with the Declaration of Helsinki.

### Reaching Task

The reaching task was conducted with an in-house parallel-link robotic manipulandum system which had two torque motors to generate the force at the end effector (Shadmehr and Mussa-Ivaldi, 1994) and one force sensor at the end effector (i.e., the handle) to measure the hand force that the participants generated to push the handle. A PC monitor was vertically mounted above the top of the robot system, approximately at the eye level of the participant. The participants sat on a chair in front of the robotic manipulandum, grasped the handle with their right hand, looked at the computer screen, and were asked to make reaching movements on the horizontal plane. The PC monitor screen provided the hand position’s online feedback as a white-filled circle with a 1-cm radius. The start position, a blue 1 cm circle, was set in front of the participants on the sagittal plane of their body. The target, a red 1 cm circle, was also on the same plane but located 10 cm apart from the start position in the forward direction. The participants were asked to maintain their hand at the start position for a randomly selected inter-trial-interval (0.5–1 s and to make a straight reaching movement towards the visual target as soon as it appeared with their own comfortable speed. In this study, movement onset was defined as the time when 5% of the maximum-velocity exceeded.

### Force Field

During perturbation trials (TR), the participant’s hand was perturbed by force generated by the robotic manipulandum, which was proportional to the movement velocity, i.e., the velocity-dependent curl force field (Shadmehr and Mussa-Ivaldi, 1994):

(1) $$\left[\begin{array}{c} f_{x}\\ f_{y} \end{array}\right]=\left[\begin{array}{cc} \text{0} & b\\ -b & \text{0} \end{array}\right]\left[\begin{array}{c} v_{x}\\ v_{y} \end{array}\right]$$

where f*x*, f*y* represent the perturbation force and ν*x*, ν*y* the velocity of the handle (i.e., the participant’s hand). In this study, perturbation was counter-clockwise (*b* = −12 Ns/m). Note that the *x*–*y* Cartesian coordinates here are on the horizontal plane and that the *y* axis connects the start and the target position.

### Error-Clamp Trials

During error-clamp trials, the robotic manipulandum generated the force channel, i.e., the stiff wall along the line connecting the start and the target positions, which restricted the hand trajectory to a straight line. The force channel clamped the trajectory error, and, thus, the trajectory error was unnoticeable to the participants (Scheidt et al., 2000; Smith et al., 2006). Since the velocity and acceleration of all movements along the x-axis (perpendicular to the channel) were minimized, the channel force enables us to measure the hand force independent of the inertial force. This perpendicular force in the channel indicated the participant’s motor commands for compensating the given force perturbation.

### Probe Trials and Error-Sensitivity

We adopted the previously developed probe trials and the corresponding estimation methods for quantifying the error-sensitivity (Herzfeld et al., 2014). The probe was composed of a triplet of a pre-perturbation error clamp trial, a perturbation trial, and a post-perturbation error clamp trial. When the force field trial was at the *n*-th trial, we measured the trajectory error *e*^(n)^ (i.e., the maximum perpendicular deviation from the straight line). In the channel trial, we measured the force *f*_x_(*t*) generated against the channel wall to compensate for the perturbation, which was perpendicular to the movement direction. Suppose the ideal force to compensate 100% of the given perturbation force is $f*\left(t\right)=b\dot{y}\left(t\right)$ where $\dot{y}\left(t\right)$ is the movement velocity along the straight line. We estimated the parameters *k*_0_, *k*_1_ to minimize (*f*(*t*) − *k*_1_f*(*t* − *k*_0_))^2^ and defined the motor memory to be updated as the parameter *k*_1_, ratio of the ideal force with respect to the generated force: *u*^(*n*)^ = *k*_1_. The forgetting factor $\alpha =\frac{u^{\left(n\;+\;1\right)}}{u\left(n\right)}$ was estimated from the data sequence of the memory *u* during error-clamp trials after probe trials. At the *n* − 1-th and *n* + 1-th trials of the error-clamp condition, we estimated the motor output *u*^(*n* − 1)^, *u*^(*n* + 1)^ as a ratio of the generated force to the ideal force for compensating the perturbation force. Thus, the error-sensitivity η^(*n*)^ was defined as,

(2) $$\eta^{\left(n\right)}=\frac{u^{\left(n\;+\;1\right)}-\alpha^{2}u^{\left(n\;-\;1\right)}}{e^{\left(n\right)}}$$

### Task Schedule and Groups

First, all subjects started with a familiarization session composed of 50 trials of reaching movement practice in the Null environment. After this familiarization, they performed 10 cycles of 13 trials: one cycle was composed of a probe trial which consisting of a triplet of a pre-perturbation error clamp trial, a perturbation trial, and a post-perturbation error clamp trial, followed by five error-clamp trials, and then another five Null (FF-Null group) or error-clamp (FF-Channel group) trials. Thus, in the FF-Null group, the trajectory error during the last five trials of one cycle was noticeable, whereas, in the FF-Channel group, it was clamped and unnoticeable. We measured the error-sensitivity *η* with the probe trials and the forgetting factor with the subsequent five error-clamp trials.

### Consistency Index of Perturbations

To quantify the force environment’s consistency, we computed the probability that the sign of the current perturbation was equal to that of the previous perturbation:

(3) $$\begin{array}{c} \mathrm{Pr}\;\left(b^{\left(n\right)}=b^{\left(n\;-\text{ 1}\right)}\right)=z\\ \mathrm{Pr}\;\left(b^{\left(n\right)}=-b^{\left(n\;-\text{ 1}\right)}\right)=\text{1}-z \end{array}$$

where *b*(*n*) is the viscosity parameter of the force field at the *n*-th trial in equation (1), as adopted from a previous article (Herzfeld et al., 2014). In addition to this measure, we also computed the lag one autocorrelation as another measure of consistency (Gonzalez et al., 2014) :

(4) $$Consistency\;:\;R\left(\text{1}\right)=\frac{E\left[\left(FF_{n}-\mu_{FF}\right)\left(FF_{n\;+\text{ 1}}-\mu_{FF}\right)\right]}{\sigma_{FF^{2}}}$$

where *FF_n_* was the perturbation force at trial *n*, *FF*_*n* + 1_ was that at trial *n* + 1, and *μ_FF_*, *σ_FF_* are the average and the variance of perturbations, respectively.

### Statistical Analysis

We applied the Mann-Whitney *U* test to reject the null hypothesis that the measured aftereffect errors (i.e., the maximum error after the five error clamp trials) of two groups (FF-Null and FF-Channel) were sampled from the same group after we confirmed that this data does not satisfy normality by the Shapiro–Wilk test. For this analysis, we used SPSS24 (IBM, USA). To examine the trend of the error sensitivities (η in Equation 2) over trials, we applied the linear mixed effect model analysis (Laird and Ware, 1982), an extension of the simple linear model analysis to allow both fixed and random effects, with the group (GR) and the trials (TR) as the fixed effects and the participants (IND) as the random effect. We supposed that the data *η* vary across participants. Besides, since our primary interest is the group difference in the trend between the two groups, we included the interaction term in this model. Using this analysis, we can estimate the within-participant effects of GR, TR, and the interaction between them. This analysis was performed with GLM function in the Statistics and Machine learning toolbox of MATLAB (MathWorks, USA) with the notation: *η* ~ 1 + GR + TR + GR * TR + (1|IND). The found interaction was further examined by the posthoc comparison of two groups for each trial to see which trial was influential in leading the interaction between trials and the groups, by the paired *t*-test with Bonferroni correction of the significant level.

## Result

**Figure 1:**
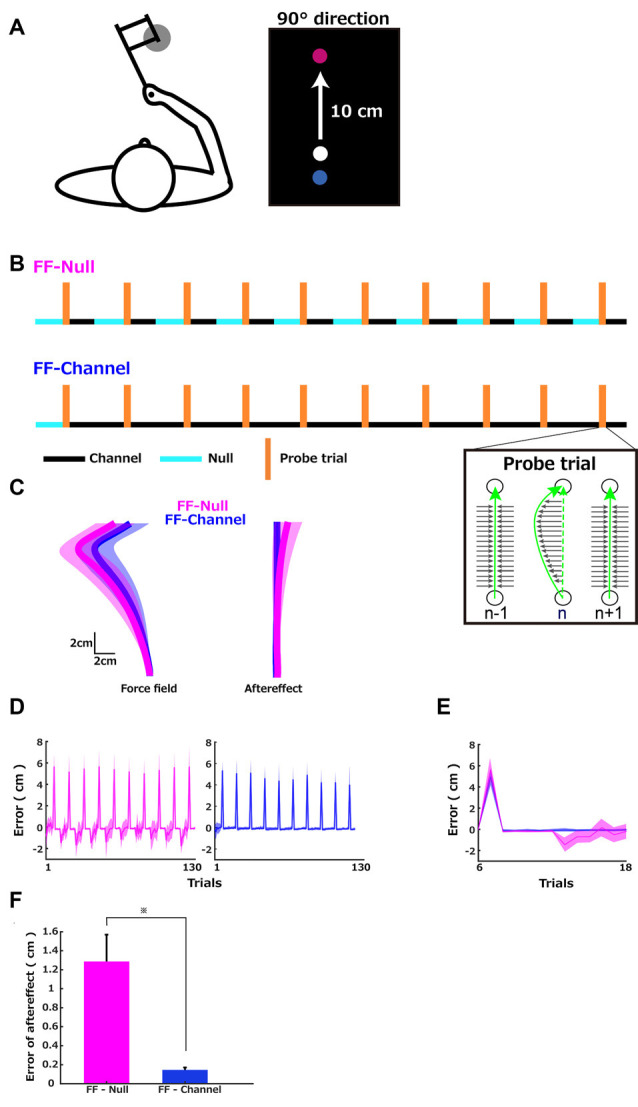
Sequences of the force environments and errors. **(A)** Left: the configuration of the reaching task with the robotic manipulandum. Right 10 cm reach task. The red circle is the target position, and the blue circle is the start position. The white circle presented the cursor position. **(B)** Sequences of the force environments of the two groups. Three different force environments were presented to the participants: a zero-force environment (Null), a velocity-dependent curl force field (FF), and a channel-force (Channel) environment. In the error-clamp trials (TR), the stiff wall constrained the trajectory to a straight line and clamped any trajectory errors. The probe trials were composed of a first error-clamp trial, a force field (FF), and a second error-clamp trial. After five Null trials as familiarization, both groups performed 10 cycles of 13 trials composed of one probe trial, five error-clamp trials, and then five error-clamp (FF-Channel) or five Null (FF-Null) trials. **(C)** Group averages of the hand trajectories. Left: (1) average of the 10 FF trials computed for each participant and average (solid line) and standard deviation (shadow) across participants in each group (FF-Null in magenta and FF-Channel in blue). Right: (1) average of the 10 sets of each cycle’s last five trials and average and standard deviation across participants. **(D)** Each trial’s maximum trajectory errors were plotted over the FF-Null (left) learning trials and FF-Channel learning trials (right). The maximum error was defined as the maximum deviation from the straight line. **(E)** The average (solid line) and SD (shadow) across participants of each trajectory’s maximum deviation plotted over trials in the first cycle. **(F)** The aftereffect in the FF-Null (magenta) and FF-Channel (blue) conditions were captured by the maximum deviation at the first trial after the five error clamp trials following the probe trial. The error bar indicates the standard error. An asterisk indicates *p* < 0.05 by Mann–Whitney *U* test.

Figure 1A left illustrates the configuration of the reaching task with the robotic manipulandum, which introduces force perturbations during 10 cm reach (Figure 1A, right), following different sequences for groups shown in Figure 1B. During the experiment, the participants held the handle of the robotic manipulandum while sitting on the chair and looking at the visual feedback presented on the vertical computer monitor. The start position was in front of the participant’s body, and the target position was always at the same center location (forward direction; 90 degrees from the horizontal line) 10 cm away from the start position. The speed of the movement measured in the channel trials was 605 ± 156 mm/s (Mean ± SD across participants) in the FF-Null group and 543 ± 130 mm/s in the FF-Channel group (*z* = 0.85, *p* = 0.39, Mann–Whitney *U* test).

### Task Consistency

Since the two sequences had the same level of consistency of perturbations (*z* = 0.85 and *R*_(1)_ = −0.08), the learning speed (i.e., the error-sensitivity) of these two groups should be equal, according to the theory of the effect of environmental statistics on learning speed (Gonzalez et al., 2014). However, when we computed Harzfeld’s *Z*-value which captures the variability of the sign of the error proposed in the previous article (Herzfeld et al., 2014), these values were 0.638 ± 0.066 (Mean ± SD) in the FF-Null group and 0.77 ± 0.064 in the FF-Channel group, which were significantly different (*z* = 3.2221, *p* < 0.001, Mann–Whitney *U* test). Thus, two theories (FF-Null group and FF-Channel group) predict different error-sensitivity profiles over the presented training schedule.

### Trajectory Error

Figure 1C left shows the group average of reach trajectories in FF trials (Mean ± SD, FF-Null group = 5.40 ± 1.21 cm, FF-Channel group = 4.94 ± 0.78 cm), illustrating a deviation from the straight line to the left because of the introduced FF perturbations. Figure 1C right shows the average across participants of 10 data sets of the last five trials of each cycle with the standard deviation. This illustrates the deviation from the straight line but toward the opposite direction, compared to the FF trials, which indicates a moderate aftereffect of the memory update in response to the exposed trajectory errors during the previous trial. Figure 1D shows the maximum deviation from the straight line throughout the training trials (the leftward error is positive). Figure 1E illustrates each trajectory’s maximum error plotted over the trials in one cycle (the first cycle composed of 6th–18th trials) of both groups. In the FF-Null group, the positive (i.e., leftward) error was generated during the FF trial, and the negative (i.e., rightward) error was generated during the five Null trials after the five error-clamp trials, thus leading to the observation of the aftereffect (Figure 1E, magenta). In the FF-Channel group, the FF trial’s error was evident, but the aftereffect of the memory update was clamped by the channel force (Figure 1E, blue). This difference in the two groups’ errors was confirmed by comparing the deviation at the first trial after the five error clamp trials that followed the probe trial between the two groups (Figure 1F). This comparison revealed a significant difference in the amplitude of the aftereffect (Mean ± SD, FF-Null group = 1.29 ± 0.84 cm, FF-Channel group = 0.15 ± 0.08 cm, Mann–Whitney *U* test, *z* = −9.255, *p* < 0.001).

### Change of Force

**Figure 2:**
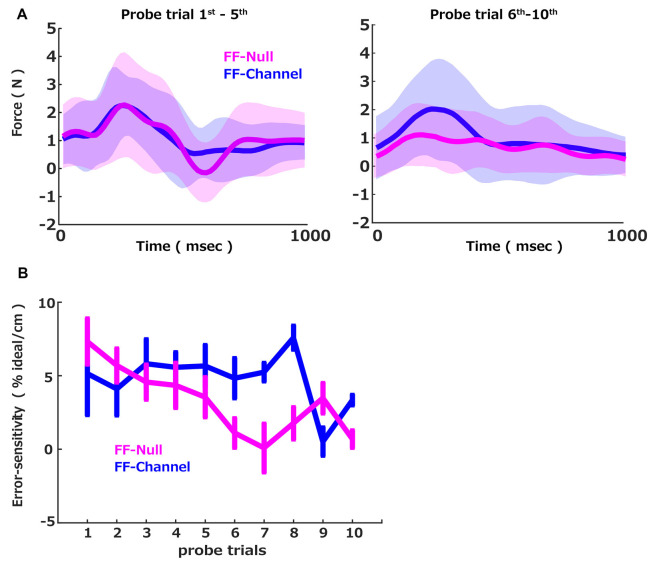
Force profiles and error-sensitivity. **(A)** The across participants average of the measured difference in the perpendicular force between the pre-perturbation error clamp trial and the post-perturbation error clamp trial. Average (solid) and standard deviation (shadow) across participants in each group (FF-Null: magenta, FF-Channel: blue) computed for the average of the first five (left) and the last five probe trials (right). **(B)** The group averages of the error-sensitivity across cycles measured by the probe trials were plotted over probe trials. The error bar indicates the standard error.

During the error-clamp trials, the participant’s hand force was measured via the force sensor mounted on the handle of the robotic manipulandum. Since the perturbation force generated by the curl force field was perpendicular to the straight line, the major component of the motor commands was also perpendicular to the straight channel force. We used a probe trial to capture the group differences of the learning speeds: a triplet of the pre-perturbation error clamp trial, the perturbation trial, and the post-perturbation error clamp trial. Since the motor command was expected to be updated by the experienced trajectory error in the perturbation trial, the perpendicular forces in the pre and post-perturbation error clamp trials were supposed to be also updated by the experienced trajectory error in the perturbation trial. Thus, the perpendicular force difference between the pre and post-perturbation error clamp trials should reflect the extent of the memory update due to the experienced errors in between these pre and post-perturbation error clamp trials. To validate this measurement, we plotted the across participants average of the measured difference in the perpendicular force between the pre-perturbation error clamp trial and the post-perturbation error clamp trial (Figure 2A; blue) separated by the first five and the last five probe trials. Note that the motion data was all aligned at the initiation of the movement (defined as the 5% of the maximum velocity) and plotted them between 0 and 1,000 ms. This profile in the difference between the pre-perturbation error clamp trial and the post-perturbation error clamp trial indicates that the generated force was updated positively by the experience of the perturbation. Also, since the presented FF was velocity-dependent, the learned compensation force was proportional to the movement speed, illustrated by a smooth bell-shaped profile (Abend et al., 1982; Flash and Hogan, 1985), which suggests that the updated motor commands were to compensate the given velocity-dependent force perturbation.

### Error-Sensitivity

We then computed how much the force was updated concerning the size of the error (i.e., error-sensitivity) and plotted it for each probe trial (Figure 2B, blue). We sought the interaction between the group and the probe trial number to examine the group difference in the error-sensitivity trend over the probe trials. To this end, we applied the linear mixed effect model with the group, the probe trials, and the interaction between the group and the probe trial number as the fixed effect and the participant as the random intercept. This analysis revealed that the probe trial number influenced the error-sensitivity, and this influence was different between the two groups. In contrast, the group itself did not influence the error-sensitivity on average. More precisely, there were significant effects of the probe trial number (*t* = −3.11, *p* = 0.002) and interaction between the probe trial number and the group (*t* = 1.98, *p* = 0.048), while no significant effect of the group (*t* = −0.58, *p* = 0.56). We further confirmed that the normality of the residual was not rejected by the Shapiro–Wilk test (W = 0.99, *p* = 0.22). Thus, we conclude that the error-sensitivity was changed over the trials differently between the FF-Null and FF-Channel groups. We further examined how the error-sensitivity was changed over the training. *Post hoc* tests (Bonferroni-corrected) showed that error-sensitivity of FF-Null was significantly lower than FF-Channel in the 6th, 7th, 8th, and 10th probes (*p* = 0.049, *p* = 0.012, *p* = 0.001, and *p* = 0.003). Here, the alpha threshold for Bonferroni-corrected was set to 0.001. Thus, the exposure to the aftereffect attenuated the error-sensitivity. Note that the effect of the aftereffect exposure was occasionally lost in the 9th probe. Nevertheless, we see the group difference in the interaction term in the linear regression analysis shown above. This indicates that the accumulated effects of the aftereffect exposure, i.e., the slope in Figure 2B, are distinct between the two groups. Thus, we conclude that the exposure to the aftereffect accumulatively suppresses the error-sensitivity.

## Discussion

We considered that two types of errors, externally induced errors and self-generated errors, may influence learning speed differently. The previous reports concluded that the statistics of externally induced errors influenced learning speed so that the consistency in the error direction and perturbations modulated error-sensitivity (Herzfeld et al., 2014). However, the effect of the self-generated errors has not been examined yet. In this study, we manipulated the self-generated errors experimentally and found that the exposure to the self-generated errors attenuated the error-sensitivity. Specifically, when the participants observed the self-generated errors which were induced by the aftereffect of memory update, the error-sensitivity that characterized the extent of the learning was attenuated to minimize the self-generated errors. On the other hand, this error-sensitivity attenuation was absent when these errors generated by the aftereffect were clamped. Combining these two observations, we conclude here that the exposure to the self-generated errors during the aftereffect of memory updates may modulate error-sensitivity and, eventually, learning speeds.

Importantly, we measured the error-sensitivity based on the validated method proposed in previous articles (Herzfeld et al., 2014). In this method, participants were presented with a triplet of trials consisting of a pre-perturbation error clamp trial, a perturbation trial, and a post-perturbation error clamp trial. By comparing the participant’s motor commands between the first and the second error-clamp trials, we confirmed that their motor commands were updated in response to the trajectory errors induced by the force perturbation (Figure 2A), which is in line with previous reports (Herzfeld et al., 2014). Although the perturbation was applied strictly every 10 trials, the extent of predictability of the perturbation between the two groups was the same. Thus, by taking the contrast of the estimated error-sensitivities between the two groups, we concluded that clamping of the aftereffects was a significant factor that modulated error-sensitivity.

According to the previously proposed theory (Herzfeld et al., 2014), any error which flips its sign with high frequency may attenuate the error-sensitivity. The other theory suggests that the perturbation statistics (e.g., uncertainty) are factors for the brain to modulate the learning speeds (Gonzalez et al., 2014). Since both studies manipulated the perturbation sequences, which also lead to a change in the error statistics, these experiments can not disassociate the two theories. On the other hand, our experiment successfully dissociated the error effects from the perturbation effects on the learning speed modulation by clamping errors. More precisely, since the perturbation schedule was the same between the two groups, the perturbation uncertainty hypothesis can not explain the group difference in the learning speed modulations over trials. This supports the idea that the perception of the errors, not the perturbation, is the factor to modulate the learning speeds. One of the novelties of our article is this point. Besides, while the error was induced by the perturbation in the previous study (Herzfeld et al., 2014), the experienced errors in our experiments were self-generated by the participants. Thus, another novel finding of this study is that exposure to the self-generated error can modulate the learning speeds.

However, there are limitations in our study for understanding the mechanism behind the effect of the error-exposure to the learning speed modulations. One major limitation of our study is that our experiment does not clarify whether the error-sensitivity statistics attenuate the error-sensitivity or whether the observation of self-generated errors attenuates the error-sensitivity no matter what the statistics of the error were. This limitation is because we did not control the sign of the error as an independent variable. The statistics of the sign of error varied across participants and trials, although it looks relatively constant on average, as shown in Figure 1E. The computed *Z*-value of our study was 0.77 ± 0.064, which is supposed to enhance the error sensitivity according to the previous work (Herzfeld et al., 2014). Since we found that the exposure to the self-generated errors attenuated the learning speeds even though the exposed error had such a high *Z*-value, we speculated that the self-generated errors affected a different learning modulation mechanism from one driven by the statistics of errors. However, further studies are requested to clarify this point. Despite this limitation, we considered that our finding of the contribution of self-generated errors on the learning speed modulation and the dissociation between the errors and the perturbation statistics might correct the previously proposed theory.

The other previous theory (Gonzalez et al., 2014) showed that the perturbation experience over multiple times might facilitate the learning, which was absent in our FF-Channel group. One possible reason for this is that our experiment’s perturbation sequence was intermittent, not consecutive. Thus, the sustainability of the effect of the perturbation experience on the learning rate modulation might not be enough to modulate learning rates with these periodic perturbations.

Acceleration of learning speeds over training sessions (i.e., savings) has been reported in the earlier reports (Krakauer et al., 1999; Kojima et al., 2004; Burge et al., 2008; Huang et al., 2011; Leow et al., 2012). Especially in one recent report, this acceleration was evident only when the perturbation direction was consistent across many training trials (Gonzalez et al., 2014). One of the statistical measures used in previous research is the Z-index, which defines the probability of a change in perturbation (Herzfeld et al., 2014). This index was equally high in both groups (FF-Null and FF-Channel) in the present experiments (*z* = 0.85 regarding perturbation sign), leading to the prediction that the error-sensitivity, i.e., learning rates, of both groups would increase equally. However, we did not find an increase in our participant’s learning speed when following such a consistent perturbation schedule. This suggests that the mechanism underlying the regulation of learning speed cannot be captured by the Z-index alone and that other factors influence the regulation of error-sensitivity.

In addition to the absence of an increase in both group’s error-sensitivity, we found a significant interaction of group and trial on the error-sensitivity. Whereas the FF-Channel group experienced only one type of error induced by the perturbations, the FF-Null group experienced the other type of error generated by their own motor commands, which were updated in response to the given perturbation. Thus, the exposure to the self-generated errors attenuated the participant’s error-sensitivity during memory updates; this indicates that the perturbation statistics are not the only factor underlying error sensitivity modulations.

The computation of the error-sensitivity, where the force update was divided by the error (Equation 2), was unstable. For example, the error-sensitivity becomes an infinite number if the measured trajectory error, the denominator of this equation, was occasionally zero. This situation of zero error or a tiny error in the perturbation trial potentially happens by participant’s trial-to-trial variability in the motor commands in the perturbation trial and the arm and hand’s very high stiffness. For instance, in or data, it appears that the effect of the aftereffect exposure was occasionally lost in the 9th probe. While we investigated the force profiles and error profiles in the 9th probe trials, we did not find any clue that there is a systematic cause of this reduction of the effect at the 9th probe trial. We considered that this reduction was occasionally observed due to instability in the computational method.

After the motor memories formed during the first training session of a motor adaptation task have been washed out, when the learner revisits the same task, the learning speed often exceeds that of the initial learning session, a phenomenon that is called “savings” (Medina et al., 2001). One possible mechanism behind this is that the recall process becomes more efficient as the brain experiences the recall of the same memory multiple times (Oh and Schweighofer, 2019). If the memory recall strongly influences the modulation of error-sensitivity, one may expect error sensitivity to increase across sessions. In our task, both groups of participants experienced the same perturbation 10 times. Although the perturbation was not applied consecutively, and thus every single perturbation might not be a strong trigger for the brain to recall the memory, it is still possible that these perturbations lead to the recall of memories. Importantly, this condition for the brain to use the perturbations as a trigger to recall memory was consistent between the two groups (*z* = 0.85 and *R*_(1)_ = −0.08). Nevertheless, we observed different profiles of the error-sensitivity change over the trial between the two groups. Thus, recall of perturbation is not the only factor underlying variations in error-sensitivity modulations.

How do self-generated errors attenuate error-sensitivity? Any kinds of error signals are inherently aversive for humans. For instance, errors in action selection induce a defensive startle response, which is the signature of an aversive response to an error (Hajcak and Foti, 2008). This error also induces the error-related negativity (ERN) in the EEG signal, which is generated in the anterior cingulate cortex (Jackson et al., 2015). Importantly, during a reaching task, the ERN induced by the trajectory errors shares the same source of the signal in the brain as the feedback-related negativity (FRN) induced by motivational error signals, which further suggests that the processing of error-based motor learning and that of motivational signals share the same neural basis (Torrecillos et al., 2014). This then leads to the hypothesis that the trajectory errors induced by the aftereffect of motor learning are also aversive. One possible way to avoid these aversive signals is to attenuate motor learning by reducing memory updates’ error-sensitivity. We speculate that error-sensitivity in the FF-Null condition was decreased to avoid the aftereffects of memory updates. On the other hand, in the FF-Channel condition, since the aftereffect was clamped, there were no demands for the brain to attenuate the memory update. This also provides a reason why learning can be accelerated in a typical motor adaptation task. When the perturbation is induced constantly, the error is generated by the perturbation, and the memory updates in response to the observed error in a certain trial decrease the error in the following trials. Thus, increasing the error-sensitivity is a rational approach to avoid the error. However, when the perturbation often switches, the memory update in response to, for instance, a rightward perturbation increases the error in the following trial with a leftward perturbation. Thus, attenuating the error-sensitivity is ecologically rational when faced with random perturbation. We, therefore, argue here that the aversiveness of errors is the crucial factor underlying the regulation of learning.

Another limitation of our study is that, although we revealed the effect of the perception of self-generated errors on motor learning, neural as well as computational mechanisms behind such an effect of perception on the implicit learning mechanism are unclear. This issue should be addressed in future work. Further, it is not yet clear how the cognition of errors influences learning rates. To examine this mechanism, a new paradigm of the experiment should be developed where the averseness of errors is directly controlled.

## Data Availability Statement

The raw data supporting the conclusions of this article will be made available by the authors, without undue reservation.

## Ethics Statement

The studies involving human participants were reviewed and approved by the Ibaraki Prefectural University of Health Sciences Review Board. The patients/participants provided their written informed consent to participate in this study.

## Author Contributions

KT, JI, SY, AY, and YK: conceptualization. KT and JI: methodology and investigation. KT and DI: formal analysis. KT and JI: writing—original draft. JI, DI, SY, AY, and YK: writing—review and editing. All authors contributed to the article and approved the submitted version.

## Conflict of Interest

The authors declare that the research was conducted in the absence of any commercial or financial relationships that could be construed as a potential conflict of interest.
